# Effects of Chlorpheniramine Maleate on Catheter-Related Bladder Discomfort in Patients Undergoing Ureteroscopic Stone Removal: A Randomized Double-Blind Study

**DOI:** 10.7150/ijms.53043

**Published:** 2021-01-01

**Authors:** Chi-Bum In, Seok-Jin Lee, Tae-Yun Sung, Choon-Kyu Cho, Young Seok Jee

**Affiliations:** 1 Department of Anesthesiology and Pain Medicine, Konyang University Hospital, Konyang University College of Medicine, Daejeon, Republic of Korea; 2 Department of Anaesthesiology and Pain medicine, Konyang University Hospital, Myunggok Medical Research Center, Konyang University College of Medicine, Daejeon, Korea

**Keywords:** catheter-related bladder discomfort (CRBD), urinary catheterization, chlorpheniramine maleate

## Abstract

Catheter-related bladder discomfort (CRBD) associated with intraoperative urinary catheterization is a distressing symptom during recovery from anesthesia. Anticholinergics have been used to manage CRBD. Chlorpheniramine maleate (CPM) is a first-generation antihistamine, which also has anticholinergic effects. This study was undertaken to evaluate the efficacy of CPM in preventing CRBD. Seventy-six adults (19-65 years old) with American Society of Anesthesiologists physical status I, II, or III of either sex, undergoing elective ureteroscopic stone removal under general anesthesia were randomized into one of two groups (each *n* = 38). Group C (control) received a placebo, and group CPM received 8 mg of intravenous CPM before the induction of anesthesia. CRBD was assessed upon arrival in the post-anesthetic care unit at 0, 1, 2, and 6 h. The severity of CRBD was graded as none, mild, moderate, and severe. Tramadol was administered when the severity of CRBD was more than moderate. The incidence rate and overall severity of CRBD did not differ between the groups at any of the time points (*р* > 0.05). The incidence of moderate CRBD was higher in group C than in group CPM only at 0 h (26.3% vs. 5.3%, *р* = 0.025). However, fewer patients in the CPM group required rescue tramadol to relieve CRBD after surgery (31.6% vs. 60.5%, *р* = 0.011). CPM administration before the induction of anesthesia had little effect on the incidence and severity of CRBD after surgery, but it reduced the administration of tramadol required to control CRBD postoperatively.

## Introduction

Catheter-related bladder discomfort (CRBD) is characterized by an urge to void or discomfort in the suprapubic region caused by urinary bladder catheterization 1-3. Catheter-related bladder irritation can occur during the postoperative period in patients who have had urinary catheterization during various surgeries 4. This condition is extremely distressing to the patient who is awakening from anesthesia and it may exacerbate postoperative pain and agitation 2.

The symptoms are similar to those of overactive bladder (urinary frequency and urgency, with or without urge incontinence), which result from involuntary contractions of the bladder mediated by muscarinic receptors 5. Therefore, muscarinic receptor antagonists are expected to affect CRBD, and agents such as tolterodine, oxybutynin, gabapentin, ketamine, and tramadol have been reported to be effective for preventing CRBD 4,6-9.

Chlorpheniramine maleate (CPM) is a classic, first-generation antihistamine often used to relieve allergic symptoms caused by histamine 10. First-generation antihistamines are also competitive muscarinic receptor inhibitors and have anticholinergic effects 11. We hypothesized that the anticholinergic effect of CPM would reduce the severity and incidence of CRBD. This randomized, controlled trial was designed to evaluate the efficacy of CPM for CRBD in patients undergoing ureteroscopic stone removal.

## Methods

This prospective, randomized, placebo-controlled study was conducted from January 2019 through October 2019 at a single university hospital after obtaining approval from the Institutional Review Board of Konyang University Hospital, Daejeon, Korea (approval number: KYUH 2018-07-033), and acquiring written informed consent from all participants. This study was registered with the Korean Clinical Research Information Service (https://cris.nih.go.kr, permit number: KCT0003236). Patients aged 19-65 years with American Society of Anesthesiologists physical status I, II, or III who were undergoing elective ureteroscopic stone surgery under general anesthesia were included in this study. The exclusion criteria included overactive bladder (frequency of more than three times per night or more than eight times per 24 h), cognitive impairment, a neuropsychological disorder, and contraindications to chlorpheniramine (e.g., hypersensitivity to CPM, prostate hypertrophy, bladder outlet obstruction, or narrow-angle glaucoma). Patients were allocated randomly (allocation ratio 1:1) to one of two groups (control group [group C] and group CPM) with a random number table generated using online randomization software (www.randomizer.org). The patients were blinded to their own group allocation. In addition, both the anesthesiologists who performed the anesthesia (board-certified anesthesiologist) and assessed the outcome variables (anesthesiology resident) and the urologists were blinded to the patient allocation.

Patients were educated about the symptoms of CRBD at the preoperative visit. A trained anesthetic nurse who prepared the study drugs (CPM or saline) opened a non-translucent envelope, which contained the group allocation when the patient arrived in the preanesthetic holding area. All patients entered the operating room without receiving premedication. Immediately after arriving in the operating room and before preparing for routine anesthetic monitoring, the patients in group CPM received 8 mg (4 mL) of intravenous CPM (Pheniramine inj^®^; Yuhan Co., Seoul, Korea), while the same volume of saline was administered intravenously to the patients in group C. Anesthesia was induced with 2 mg/kg of propofol and 1-2 μg/kg of fentanyl, and endotracheal intubation was facilitated with 0.6 mg/kg of rocuronium. After intubation, volume-controlled mechanical ventilation was initiated and anesthesia was maintained with an oxygen/nitrous oxide mixture (50:50) and 3-8 vol% end-tidal concentration of desflurane to maintain the Patient State Index (SedLine^®^; Masimo Corp., Irvine, CA, USA) at 25-50. Urinary catheterization was performed at the end of the procedure using a Foley catheter lubricated with 2% lidocaine jelly (Instillagel^®^; Farco-Pharma GmbH, Cologne, Germany) and the balloons of the catheter were inflated with 5 mL of normal saline. The size of the Foley catheter was determined at the discretion of the urologist. All surgeries and urinary catheterizations were performed in the lithotomy position by (board-certified) urologists. After urinary catheterization, the position of the patient was changed to supine. All anesthetics were stopped and the neuromuscular block was reversed with neostigmine (50 μg/kg) and glycopyrrolate (10 μg/kg). Extubation was performed using the same extubation criteria in both groups: spontaneous respiratory breathing (10-25 rate/min), tidal volume ≥ 5 mL/kg, response to verbal commands, and neuromuscular train-of-four (TOF) by acceleromyography (TOF-Watch SX^®^; Organon Ltd., Dublin, Ireland) on the adductor pollicis muscle ≥ 0.9. The time interval from discontinuation of the anesthetics to eye-opening and extubation was recorded during emergence from anesthesia. All patients were transferred to the post-anesthetic care unit (PACU) after removing the endotracheal tube.

The primary outcomes of this study were the incidence of CRBD at 0, 1, 2, and 6 h after the patient arrived in the PACU. The secondary outcomes were the severity of CRBD, the number of patients who required rescue medication (tramadol) for relief of CRBD symptoms, and adverse events. The incidence and severity of CRBD were evaluated using a 4-point scale as follows 0, 1, 2, and 6 h after arrival in the PACU by the anesthesiology resident: none = no complaints of any CRBD symptoms even when asked about them, mild = reported by the patient only on direct questioning, moderate = spontaneous complaint by the patient of CRBD symptoms without any behavioral responses (e.g., attempts to pull out the catheter, flailing limbs, or a loud vocal response), and severe = spontaneous complaint by the patient of CRBD symptoms with behavioral responses 8. CRBD was considered to be present when the scale was evaluated as mild, moderate, or severe. We defined symptoms of CRBD as bladder discomfort, such as the urge to urinate and discomfort in the suprapubic region, and distinguished it from postoperative pain. Postoperative pain was assessed using a numerical rating scale (NRS) score (0 = no pain; 10 = worst pain imaginable). All patients were observed in the PACU for at least 1 h after surgery and the highest NRS score evaluated in the PACU was recorded. If the CRBD was rated moderate or severe in the PACU or ward, 50 mg of tramadol was administered intravenously, and if the NRS for surgical pain was ≥ 4, 25 mg of pethidine was administered intravenously. If the pain NRS was ≥ 4 and CRBD was moderate or severe, only 50 mg of tramadol was administered without pethidine. The Ramsay Sedation Scale (RSS: 1 = patient anxious, agitated, or restless; 2 = patient cooperative, oriented, and tranquil alert; 3 = patient responds to commands; 4 = asleep, but with a brisk response to a light glabellar tap or loud auditory stimulus; 5 = asleep, with a sluggish response to a light glabellar tap or a loud auditory stimulus; and 6 = asleep, with no response) 12 was also applied when CRBD was evaluated. A RSS score ≥ 4 was defined as sedation. In addition, all adverse events during the first 6 h postoperatively were recorded and analyzed.

Based on a previous study 13, 70% of patients receiving urinary catheterization complain of postoperative CRBD. A sample size of 36 patients per group was required to detect a 50% reduction in the incidence of CRBD with a power of 0.8 and an α-value of 0.05 (two-sided). Considering a potential dropout rate of 5%, 38 patients per group were included. The statistical analysis was performed using SPSS software (ver. 18.0 for Windows; SPSS Inc., Chicago, IL, USA). The distribution of continuous variables was assessed with the Kolmogorov-Smirnov test. Continuous variables were analyzed using Student's *t*-test or the Mann-Whitney U test, as appropriate. Categorical variables (e.g., incidence and severity of CRBD and adverse events) were analyzed using the χ^2^ test, χ^2^ test for trends (linear-by-linear association), or Fisher's exact, where appropriate. A *р*-value < 0.05 was considered significant for all analyses.

## Results

**Figure 1:**
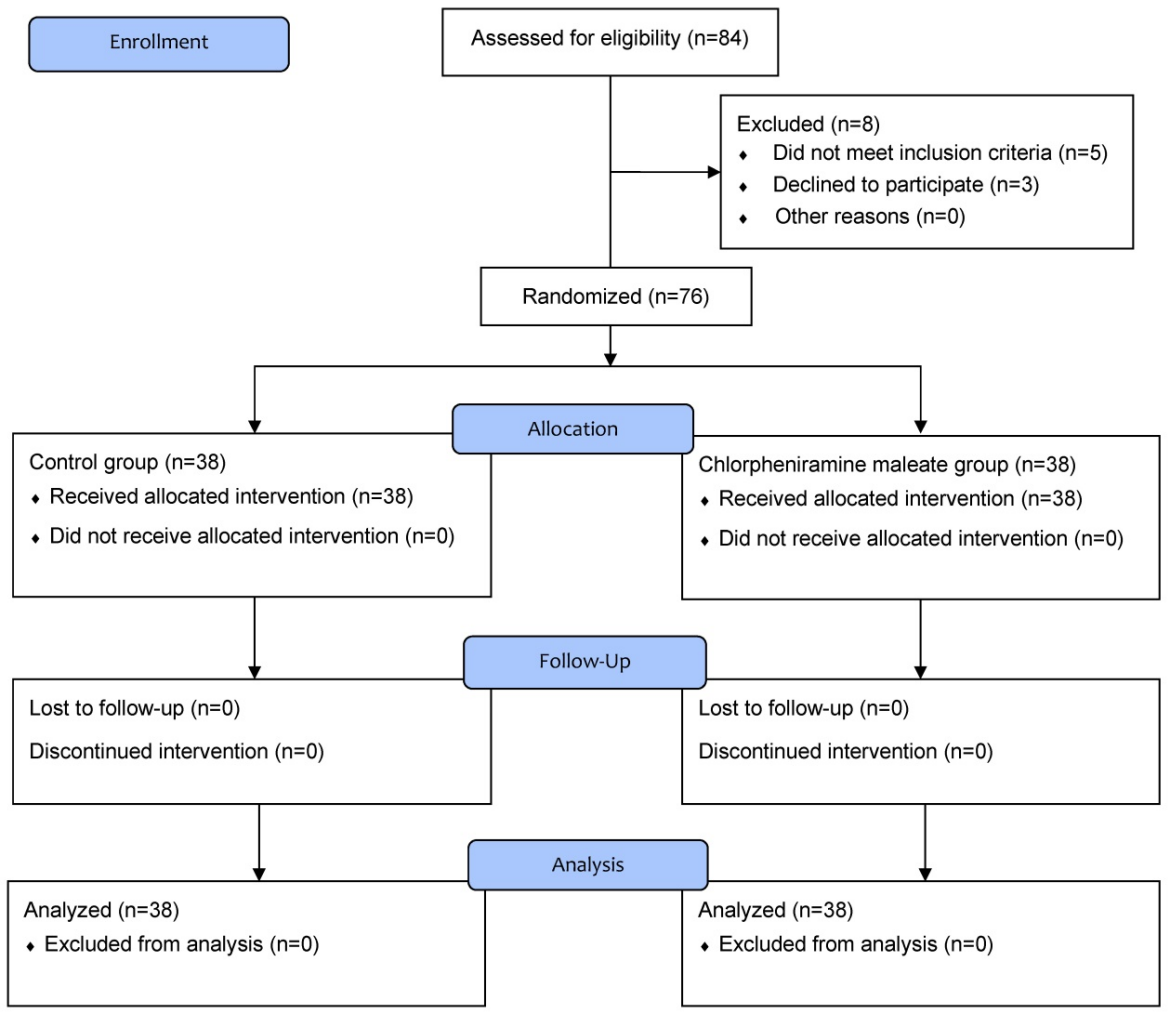
Flow chart of patient enrollment.

In total, 84 patients were screened for eligibility and 8 were excluded; 2 patients had overactive bladder, 2 patients had a neuropsychological disorder, 1 patient had prostate hypertrophy, and 3 patients refused to participate in the study. Consequently, 76 patients were randomly allocated to group C or CPM and completed this study (Figure 1).

No differences were observed in the characteristics of the patients between the two groups (Table 1).

The incidences and overall severity of CRBD, which were assessed 0, 1, 2, and 6 h after the patient arrived in the PACU, were comparable between the two groups. However, the incidence of moderate CRBD was higher in group C than in group CPM in the CRBD evaluation at 0 h (26.3% vs. 5.3%, *р* = 0.025) (Table 2).

Recovery and postoperative data are shown in Table 3. The time interval from discontinuation of anesthetics to eye-opening and extubation was similar between the groups. Postoperative pain NRS and requirements for pethidine were not different between the groups. However, the dose of tramadol administered was higher in group C (median [interquartile range], 50 [0-50] vs. 0 [0-50], *р* = 0.009) and more patients in group C needed tramadol compared to those in group CPM (60.5% vs. 31.6%, respectively, odds ratio 3.332, 95% confidence interval 1.3-8.5, *р* = 0.011). Also, the number of tramadol administrations differed between the groups (*р* = 0.011).

Adverse events were comparable between the groups (Table 4).

## Discussion

In this study, we observed that administration of a single dose of intravenous CPM (8 mg) before the induction of anesthesia did not reduce the severity or incidence of postoperative CRBD in patients who underwent ureteroscopic stone removal. However, CPM reduced the total usage of postoperative tramadol, with no differences in drug-related side effects between the groups.

The urinary tract has three sets of innervation: sacral parasympathetic, thoracolumbar sympathetic, and sacral somatic nerves (mainly pudendal nerves) 14. The urinary bladder receives parasympathetic innervation from the pelvic nerve and sympathetic innervation from the hypogastric nerve 8. Contraction of the bladder involves detrusor activity, which is mediated by muscarinic receptors located in the bladder wall stimulated by acetylcholine released from activated parasympathetic nerves 9. There is a heterogeneous population (M1-5) of muscarinic receptor subtypes. Among them, the M2 muscarinic receptor subtype is predominant in the bladder, but a smaller population of M3 receptors mediates its contraction 15,16.

To properly manage CRBD, it is necessary to distinguish it from postoperative pain. Because the mechanisms underlying CRBD and postoperative pain differ, CRBD is resistant to conventional pain management, such as opioids 2,13. In this study, the synthetic opioid pethidine was used as a rescue analgesic for postoperative control of the pain; it is very unlikely that this affected CRBD.

In previous studies, antimuscarinic agents such as tolterodine 4 and oxybutynin 6 have been reported to be effective for preventing and treating CRBD. In addition, several types of agents, including the anticholinergics (glycopyrrolate 17 and butylscopolamine 1), the antiepileptics (gabapentin 7 and pregabalin 18), the anesthetics/sedatives (ketamine 8 and dexmedetomidine 2), and the analgesics (tramadol 9 and nefopam 19), decrease CRBD. They seem to have a preventive effect on CRBD via antimuscarinic effects, whereas side effects such as dry mouth, tachycardia, and sedation occur due to the nature of each agent.

CPM is a first-generation antihistamine and alkylamine that is widely used for symptomatic relief of allergic conditions 10,20. Its action is achieved by inhibiting H1 receptors on cells, antagonizing the reversible effects of histamine, and competitively inhibiting the target organ 21. First-generation antihistamines are also competitive inhibitors of muscarinic receptors and have anticholinergic effects 10,11,21-23. In addition, antihistamines have an analgesic effect 21,22. Peak plasma concentrations after the intravenous administration of CPM are achieved after 15 min, with plasma half-lives of 8-17 h 21. Because CPM readily penetrates the blood-brain barrier and acts on central H_1_ receptors, it has the potential to cause adverse central nervous system effects, such as sedation, somnolence, and cognitive impairment, and its antimuscarinic effects can cause symptoms, such as dry mouth and blurred vision 23.

In this study, CPM did not increase the incidence of side effects, such as sedation or dry mouth; however, it failed to decrease the severity and incidence of CRBD. Although the incidence of moderate CRBD was higher in the control group 0 h after patients arrived in the PACU, the overall incidence and severity at 0, 1, 2, and 6 h did not differ between the two groups. These results are different from those of other studies that used antimuscarinic agents to prevent CRBD. A few possible reasons may explain these results. First, our study administered intravenous CPM before the induction of anesthesia. This has the advantage that CPM can prevent perioperative anaphylaxis caused by anesthetics, such as muscle relaxants 24. Also, this approach reduces the incidence of cough that can occur due to fentanyl use during induction 25. However, considering that the peak plasma concentration of intravenous CPM is achieved in about 15 min 21, it is difficult to expect CPM to have the greatest effect during recovery after surgery. In fact, in our study, the number of patients with moderate and severe CRBD at 0 and 1 h tended to decrease compared to the control group when using CPM, but not later. We speculate that this tendency in the early stages of recovery contributed to the decrease in tramadol usage in the group using CPM. Therefore, if CPM was administered at the end of surgery, another outcome could have been possible. Second, we used 8 mg as the CPM dose. First-generation antihistamines have weak antimuscarinic anticholinergic effects 23. In addition, the anticholinergic potency of CPM is lower than that of cyproheptadine and promethazine 26. Antimuscarinic activity of CPM was observed at a higher dose in an experimental study 27. CPM may have limited efficacy for CRBD; to address this, the dose of CPM can be increased but this may lead to side effects, including adverse effects on the central nervous system and/or cardiovascular system. The usual recommended intravenous dose of CPM for adults is 5-40 mg (24 h maximum of 40 mg) 20. The therapeutic dose for anaphylactic reactions and allergic conditions in adults is 10-20 mg, but hypotension may occur after administering CPM in that dose range 28. In a study that evaluated the safety of prophylactic administration of an antihistamine after induction of anesthesia, 8 mg of CPM did not cause any clinically significant hemodynamic changes 29. Therefore, we used a CPM dose of 8 mg.

Some limitations of our study should be discussed. We evaluated the effect of a single dose of CPM on CRBD during ureteroscopic stone removal. However, we did not evaluate the dose-response titration during the postoperative period. Therefore, further research is needed to determine the dose-response relationship of CPM to CRBD. Second, we did not evaluate the response in patients undergoing different types of surgical procedures. Inserting a ureter stent after stone removal can cause suprapubic pain, which can be mistaken for CRBD. Finally, a masking effect was possible due to the use of glycopyrrolate during the study period. Glycopyrrolate is an anticholinergic agent that showed a preventive effect on CRBD 17 and may have affected the reduced severity and incidence of CRBD in the both groups. Considering that the peak activity of intravenous glycopyrrolate is 5-10 min 30, glycopyrrolate may have affected CRBD outcomes during the early stages of postoperative recovery.

In conclusion, administering intravenous CPM (8 mg) before the induction of anesthesia did not reduce the postoperative severity or incidence of CRBD in patients undergoing ureteroscopic stone removal. However, a single dose of CPM reduced the total usage of postoperative rescue tramadol for controlling CRBD during the postoperative period. Therefore, further investigation is needed to assess the effect of CPM on CRBD.

## Author Contributions

All of the listed authors were involved in the drafting of the work, approved the final manuscript, and agreed to be accountable for all aspects of this work.

1. Chi-Bum In

This author helped writing the manuscript, analyzing and interpretation of data.

2. Seok-Jin Lee, Choon-Kyu Cho, Young Seok Jee

These authors helped the acquisition, analysis, and interpretation of data.

3. Tae-Yun Sung

This author helped the conception and design of the study, statistical analysis and writing the manuscript.

## Figures and Tables

**Table 1:** Patient characteristics

	Control (*n* = 38)	Chlorpheniramine (*n* = 38)	*р-*value
Age (years)	49.8 ± 9.0	47.8 ± 12.2	0.414
Sex (male/female)	24/14	22/16	0.639
Height (cm)	162.7 ± 7.9	165.6 ± 8.8	0.128
Weight (kg)	69.7 ± 10.2	70.9 ± 15.1	0.679
Body mass index (kg/m^2^)	26.3 ± 3.1	25.7 ± 4.4	0.507
ASA classification			
I/II/III	9/27/2	9/28/1	0.815
Position of stone			
Kidney/Ureter/Both	12/20/6	12/23/3	0.587
Duration of surgery (min)	47.5 [33.8-105.0]	47.5 [25.0-75.0]	0.204
Duration of anesthesia (min)	98.9 ± 55.6	81.1 ± 32.9	0.093
Fluids (mL)	200.0 [137.5-300.0]	200.0 [150.0-300.0]	0.912
Urinary catheter size (Fr)			
14/16/18	10/26/2	11/27/0	0.485

Values indicate the mean ± standard deviation, numbers, or median [interquartile range]. ASA: American Society of Anesthesiologists.

**Table 2:** Incidence and severity of postoperative catheter-related bladder discomfort

	Control (*n* = 38)	Chlorpheniramine (*n* = 38)	*р-*value
0 h			
Incidence	18 (47.4%)	11 (28.9%)	0.098^*^
Severity			0.064^†^
Mild	8 (21.1%)	8 (21.1%)	NA
Moderate	10 (26.3%)	2 (5.3%)	0.025^‡^
Severe	0 (0%)	1 (2.6%)	> 0.999^‡^
1 h			
Incidence	25 (65.8%)	26 (68.4%)	0.807^*^
Severity			0.309^†^
Mild	12 (34.2%)	20(52.6%)	0.063^*^
Moderate	12 (31.6%)	6 (15.8%)	0.105^*^
Severe	1 (2.6%)	0 (0%)	> 0.999^‡^
2 h			
Incidence	27 (71.1%)	27 (71.1%)	1.000^*^
Severity			0.704^†^
Mild	24 (63.2%)	22 (57.9%)	0.639^*^
Moderate	3 (7.9%)	5 (13.2%)	0.711^‡^
Severe	0 (0%)	0 (0%)	NA
6 h			
Incidence	17 (44.7%)	17 (44.7%)	1.000^*^
Severity			0.551^†^
Mild	14 (36.8%)	17(44.7%)	0.484
Moderate	3 (7.9%)	0 (0%)	0.240^‡^
Severe	0 (0%)	0 (0%)	NA

Values are numbers (%). NA: not applicable. ^*^χ^2^ test, ^†^χ^2^ test for trends (linear-by-linear association), ^‡^Fisher's exact test.

**Table 3:** Recovery and postoperative data

	Control (*n* = 38)	Chlorpheniramine (*n* = 38)	*р-*value
Time to eye opening (min)	7.1 ± 2.2	6.8 ± 2.2	0.533^*^
Time to extubation (min)	7.9 ± 2.5	7.6 ± 3.4	0.605^*^
Tramadol, mg	50 [0-50]	0 [0-50]	0.009^†^
Tramadol, *n* (%)	23 (60.5%)	12 (31.6%)	0.011^‡^
Number of tramadol administration			0.011^§^
0	15 (39.5%)	26 (68.4%)	
1	17 (44.7%)	10 (26.3%)	
2	6 (15.8%)	2 (5.3%)	
NRS for pain	1.5 [0-4.25]	1 [0-2.25]	0.101^†^
Pethidine, mg	0 [0-0]	0 [0-0]	0.229^†^
Pethidine, *n* (%)	5 (13.2%)	2 (5.3%)	0.430^ΙΙ^

Values indicate the mean ± standard deviation, numbers (%), or median [interquartile range]. NRS: numerical rating scale (0 = no pain, 10 = worst imaginable pain). ^*^Student's *t*-test, ^†^Mann-Whitney U test, ^‡^χ^2^ test, ^§^χ^2^ test for trends (linear-by-linear association), ^ΙΙ^Fisher's exact test.

**Table 4:** Adverse events

	Control (*n* = 38)	Chlorpheniramine (*n* = 38)	*р-*value
Sore throat	12 (31.6%)	15 (39.5%)	0.472^*^
Hoarseness	1 (2.6%)	0 (0%)	> 0.999^†^
Dry mouth	3 (7.9%)	3 (7.9%)	NA
Nausea	3 (7.9%)	3 (7.9%)	NA
Vomiting	2 (5.3%)	0 (0%)	0.493^†^
Headache	0 (0%)	2 (5.3%)	0.493^†^
Dizziness	1 (2.6%)	1 (2.6%)	NA
Dyspepsia	1 (2.6%)	0 (0%)	> 0.999^†^
Sedation (RSS ≥ 4)	1 (2.6%)	2 (5.3%)	> 0.999^†^

Values are numbers (%). NA: not applicable; RSS: Ramsay sedation scale. ^*^χ^2^ test for trends (linear-by-linear association), ^†^Fisher's exact test.
